# The Efficacy of Traditional Chinese Medicine Shoutai Pill Combined with Western Medicine in the First Trimester of Pregnancy in Women with Unexplained Recurrent Spontaneous Abortion: A Systematic Review and Meta-Analysis

**DOI:** 10.1155/2020/7495161

**Published:** 2020-08-08

**Authors:** Hui-fang Li, Qi-hong Shen, Xiao-qing Li, Zhang-feng Feng, Wei-min Chen, Jia-hua Qian, Li Shen, Li-ying Yu, Yi Yang

**Affiliations:** ^1^Department of TCM Gynecology, Tongxiang Maternal and Child Health-Care Center, Tongxiang, Zhejiang 314500, China; ^2^Department of Anesthesiology, Affiliated Hospital of Jiaxing University, The First Hospital of Jiaxing, Jiaxing, Zhejiang 314000, China; ^3^Department of Obstetrics, Tongxiang Maternal and Child Health-Care Center, Tongxiang, Zhejiang 314500, China; ^4^Department of Gynecology, Tongxiang Maternal and Child Health-Care Center, Tongxiang, Zhejiang 314500, China; ^5^Department of Internal medicine, Tongxiang Maternal and Child Health-Care Center, Tongxiang, Zhejiang 314500, China; ^6^Department of Nursing, Tongxiang Maternal and Child Health-Care Center, Tongxiang, Zhejiang 314500, China

## Abstract

**Background:**

Shoutai Pill (STP), a famous classic herbal formula documented in traditional Chinese medicine (TCM), is widely available in China for treating unexplained recurrent spontaneous abortion (URSA). This systematic review and meta-analysis aims at evaluating the efficacy and safety of STP in the first trimester of pregnancy in women with a history of unexplained recurrent spontaneous abortion.

**Methods:**

The following eight databases were searched from their establishment to Dec 31, 2019, for randomized controlled trials (RCTs): PubMed, Embase, Cochrane Library, Web of Science, China National Knowledge Infrastructure (CNKI), the Chinese BioMedical database (CBM), Chinese Scientific Journal Database (VIP), and the Wanfang database. The quality of evidence was estimated by the Grading of Recommendations Assessment, Development, and Evaluation (GRADE).

**Results:**

A total of 12 studies (916 patients) with URSA were contained in this meta-analysis. The forest plot showed that patients treated with Shoutai Pill and western medicine had a significantly lower incidence of early pregnancy loss (RR: 0.42; 95% CI: 0.34–0.52; *P* < 0.01, *I*^2^ = 0%). Subgroup analysis revealed that different types of TCM syndrome differentiation had the similar results. Also, in the combined group, patients had a lower TCM syndromes and symptoms and serum D-dimer level, while higher incidence of live birth.

**Conclusions:**

Our findings suggest that cotreatment with STP and western medicine might be superior to western medicine alone in the first trimester of pregnancy to prevent miscarriage in women with unexplained recurrent spontaneous abortion, and there was no adverse event in the experimental group reported. However, the methodological quality of included RCTs was unsatisfactory; it is necessary to verify its effectiveness with further more standardized researches of rigorous design.

## 1. Introduction

Recurrent spontaneous abortion (RSA), which is classically defined as the occurrence of two or more clinically consecutive pregnancy losses before 20 gestational weeks in fertile couples [1], is one of the most frustrating and difficult areas in reproductive medicine with complex etiologic factors varying from genetic abnormality, endocrine disorders, abnormal anatomic structures, infectious, to immune factors [2–4] and may affect about 1% to 5% of women in reproductive age [3, 4]. However, approximately 50% of RSA remain unknown and unresolved [5], a condition commonly known as unexplained recurrent spontaneous abortion (URSA) after a diagnosis of exclusion and considered to be early spontaneous abortion when it occurs before 12 weeks [6]. Emerging evidence shows that immunological disturbances and abnormal coagulation and anticoagulation may be involved in this disorder [7, 8]. Various therapeutic strategies such as immunologic intervention with allogenic lymphocyte [9] and immunoglobulin [10], anticoagulant therapy using aspirin [11] or low-molecular weight heparin [12], hormonal supplementation with progesterone or dydrogesterone [13], and microelements supplementation, such as vitamin E, vitamin D [14], and folic acid [15], have been used to improve pregnancy outcomes among these women, but no effective treatment has been identified. To date, there is no widely accepted therapeutic approaches for URSA [16], bringing seriously physical and mental impacts to both the affected female population and their families [17–19]. Therefore, it is essential to investigate effective treatments to reduce pregnancy losses and maintain successful pregnancy preservation in patients with URSA.

In recent years, Chinese medicines have been accepted as a mainstream of medical care and have become a popular complement to western medicines in the treatment of unexplained recurrent spontaneous abortion patients, with satisfied effect and high safety and reliability [20–22]. Based on the traditional Chinese medicine theory, URSA was named as “Hua Tai” with pathogenesis being always dominated by the deficiency of the kidneys [23], which is equivalent to vital energy, stores essence, dominates reproduction, and bears a close relationship to reproductive endocrine system [24]. Patients with kidney deficiency are unable to promote blood flow leading to blood stasis and mutual regulation exists between kidney deficiency and blood coagulation [25]. Therefore, the focus of treatment is to reinforcing kidney to replenish essence, nourishing the blood and promoting blood circulation to maintain a good pregnancy [26]. Shoutai Pill (STP) is a classical herbal formula invented by a famous TCM physician Xichun Zhang in *Yi Xue Zhong Zhong Can Xi Lu* (Records of Tradition Chinese and Western Medicine in Combination), with the composition of four commonly used natural herbs: Chinese Dodder Seed, Himalayan Teasel Root, Chinese Taxillus Twig, and Donkey-hide Glue [27]. Over the past years, accumulating data from case reports, noncontrolled trials, animal experiments, and RCTs have generally reported consistent findings regarding the pregnancy outcome-heightening and symptom-improving effects of STP, either administrated alone or in combination with western medicine, for the management of URSA [28–30]. Despite its wide application, at present, it still lacks of comprehensive systematic review and meta-analyses to guidance. Here, we aim to investigate the efficacy and safety of TCM classical prescription Shoutai Pill combined with western medicine in patients with URSA based on a meta-analysis of the data from the selected studies.

## 2. Material and Methods

### 2.1. Methods

We reported this systematic review and meta-analysis following the Preferred Reporting Items for Systematic Reviews and Meta-Analyses (PRISMA) guidelines [31]. The number of registration in PROSPERO is CRD42020169875.

### 2.2. Types of Studies

All of the random controlled trials (RCTs) examining the effect of Shoutai Pill combined with western medicine for the treatment of unexplained recurrent spontaneous abortion with no limitations on language and publication status were included. Non-RCTs or animal experiments were excluded.

### 2.3. Types of Participants

Patients who had been confirmed pregnant by serum human chorionic gonadotropin (HCG) or ultrasound in the first trimester of pregnancy with a diagnosis history of URSA, defined as two or more spontaneous abortions that had ruled out the following four definite etiologies: infections, abnormal parental karyotypes, endocrine disorders, and anatomic abnormality, regardless of maternal age, gestational age, ethnicity, nationality, education, or economic status. The inclusion criteria also stipulated that there had been no treatment given before pregnancy or the entry into the trials. Trials involving recurrent spontaneous abortion with definite etiologies and URSA participants without pregnancy were excluded.

### 2.4. Types of Interventions

We included studies using the common prescription of Shoutai Pill as a basic formula and modified according to syndrome differentiation meanwhile, regardless of the dose, method of dosing, or duration of administration, in combination with western medicine compared with western medicine excluding bed rest and psychological supports. The western medicine should remain the same in the control group in the same RCT. All the treatments were initiated after the patients were confirmed to be pregnant. If trials included other cointerventions such as acupuncture, acupoint application, and moxibustion, they were excluded.

### 2.5. Types of Outcome Measures

Primary outcome is the incidence of early pregnancy loss, as defined by the authors. Second outcomes included the incidence of live birth, as defined by the authors, TCM syndromes and symptoms, serum D-dimer level, and incidence of maternal and perinatal adverse events during treatment. Studies without pregnancy outcome reported were excluded.

### 2.6. Search Strategy

We systematically conducted electronic searches in the following clinical studies databases: PubMed, Embase, Cochrane Library, Web of Science, China National Knowledge Infrastructure (CNKI), the Chinese BioMedical database (CBM), Chinese Scientific Journals Database (VIP), and the Wanfang database with no limitations on language and publication status for RCTs examining the effect of Shoutai Pill combined with western medicine for the treatment of URSA from inception of each databases through to 31 December 2019. We made the retrieval formula according to the PICOS strategy. The search terms for literature searching were as follows: (“recurrent spontaneous abortion” OR “recurrent miscarriage” OR “habitual abortion” OR “recurrent pregnancy loss” OR “hua tai”) AND (“shou tai pill” OR “shoutai pill” OR “shoutai wan” OR “shou tai wan”) AND (“clinical research” OR “randomized controlled trial” OR “randomization” OR “RCT” OR “random grouping”). We also manually searched the reference lists of all identified articles for possible related studies to supplement the relevant literature.

### 2.7. Data Extraction and Quality Assessment

Two researchers (Hui-fang Li, Qi-hong Shen) extracted the general information of the eligible studies by a predesigned and standardized data extraction form: first author, year of published, TCM syndrome differentiation, sample size, age, gestational age, times of abortions, definition of miscarriage and live birth, intervention time, treatment interventions and control groups, treatment duration, and outcomes. Any conflict was resolved by a third author (Wei-min Chen). The methodologic quality of each individual study was independently evaluated by two researchers (Li Shen, Yi Yang) in reference to the Cochrane Handbook for Systematic Reviews of Interventions. We evaluated using the following criteria: random sequence generation, allocation concealment, blinding of participants and personnel, blinding of outcome assessments, incomplete outcome data, selective reporting, and other bias. Each study was classified into low risk, high risk, or unclear. If there was a disagreement, we referred to the views of the third researcher (Wei-min Chen).

### 2.8. GRADE Evaluation

The quality of outcome was evaluated by GRADE (Grading of Recommendations Assessment, Development, and Evaluation) according to the following criteria: study design, risk of bias, rating inconsistency in results, rating indirectness of evidence, and others. The quality of evidence was classified as high, moderate, low, or very low.

### 2.9. Statistical Analysis

We conducted this meta-analysis by using Review Manager (RevMan) (computer program) (Version 5.3. Copenhagen: The Nordic Cochrane Centre, The Cochrane Collaboration, 2014). Regarding the study outcomes, relative risk (RR) with 95% confidence interval (CI) was used for binary variables, while the weighted mean difference (WMD) and 95% CI were presented for continuous variables. Cochrane's *P* values and *I*^2^ were tested to examine heterogeneity among the studies. High heterogeneity most likely existed due to the clinical and methodological factors, so the random effect model was adopted in this meta-analysis even *I*^2^ was small. Subgroup analysis was performed based on the type of TCM syndrome: kidney deficiency and blood stasis, spleen and kidney deficiency, or without reported.

Funnel plots and the Egger regression test were performed to examine potential publish bias. In addition, sensitivity analysis was performed by sequentially deleting trials to check the stability of the primary outcomes.

## 3. Result

### 3.1. Search Results

**Figure 1:**
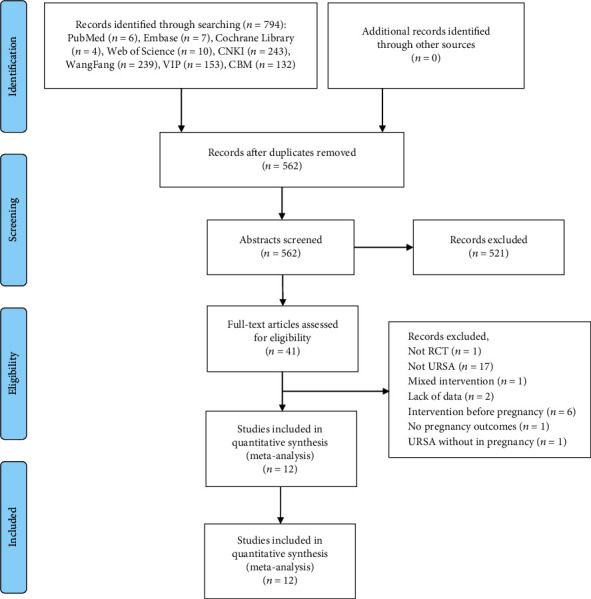
The inclusion process of literature.

Initially, a total of 794 relevant studies were collected. After excluding duplicate studies, we scanned 562 studies based on their abstracts and titles. Then, 41 articles were evaluated by full text. And 29 trials were eliminated for the following reasons: seventeen non-URSA studies, one study was not RCT, one trial without pregnancy outcome reported, one study included URSA women without pregnancy, one article with mixed interventions, two articles were lacking data, and six studies giving intervention before pregnancy. Eventually, 12 studies were included in our system review. The search process is displayed in Figure 1.

### 3.2. Study Characteristics

The basic information of the included RCTs is summarized in Table 1. Of these trials, all of them were published in China. A total of 916 patients with URSA were contained in these studies, 460 participants were assigned to the treatment group, and 456 were designated in the control group. The sample sizes of these trials ranged from 40 to 150. The baseline in all of the trials did not significantly differ. All of the trials involved two-arm designs: treatment group versus control group. Patients in the treatment group were treated with STP (Table 2) in combination with western medicine, while patients in the control group were administered with western medicine alone, including ten RCTs used natural P (progesterone injection, progesterone capsule, or dydrogesterone) [32–41], whereas the other two studies used natural P combined with other treatment, one with allogenic lymphocyte immunotherapy [42] and one with low-molecular weight heparin sodium [43]. Treatment duration varied from fourteen days to 20 gestational weeks with two articles not mentioned [35, 39]. Regarding TCM syndrome differentiation, 5 articles were kidney deficiency and blood stasis [33, 37, 39–41], 1 trial was spleen and kidney deficiency [34], and 6 studies not mentioned [32, 35, 36, 38, 42, 43]. Of the 12 trials, 11 reported the incidence of early pregnancy loss [32–42], five presented the live birth rate [32, 33, 37, 39, 43], 8 trials mentioned the TCM syndromes and symptoms [32, 33, 35, 37–40, 43], 4 trials stated serum D-dimer level [35, 37, 39, 40], and three trials mentioned the adverse events [36, 40, 41].

### 3.3. Risk of Bias Assessment

**Figure 2:**
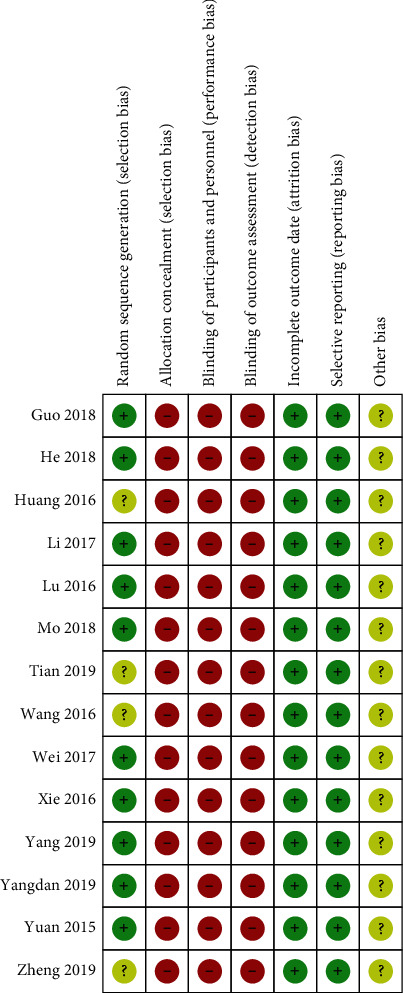
The risk of bias about each included study.

In general, the methodological quality of the included trials was poor. All of the 12 included studies involved two-arm designs and declared as random controlled trials, and 8 trials reported proper generation methods (random number table or coin toss) with a low risk of bias [32, 33, 35–37, 39–41]. Four trials did not describe the randomization procedure clearly [34, 38, 42, 43]. None of the trials reported any concealed allocation or blinding of patients and investigators. No trial indicated the number and reasons of dropouts. No selective reporting was reported. None of the included trials calculated the sample size. The results of the assessments are shown in Figure 2.

## 4. Outcome Measures

### 4.1. Primary Outcome

**Figure 3:**
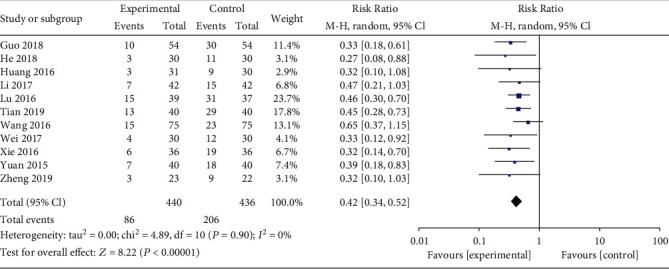
Forest plot for the incidence of early pregnancy loss between combined and western alone medicine group.

**Figure 4:**
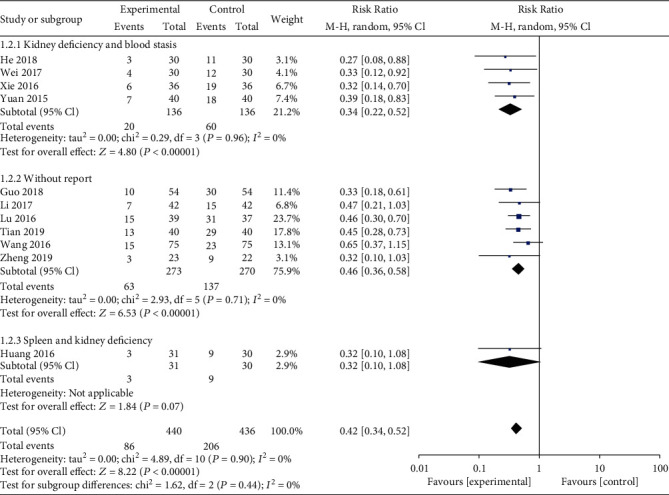
Subgroup analysis for early pregnancy loss rate based on the different types of TCM syndrome differentiation.

Eleven studies reported the incidence of early pregnancy loss, and meta-analysis showed that patients in the combined group had a significantly lower occurrence (RR: 0.42; 95% CI: 0.34–0.52; *P* < 0.01, *I*^2^ = 0%, Figure 3). Subgroup analysis revealed that different types of TCM syndrome differentiation had a lower incidence of early pregnancy loss in the combined group (Figure 4).

### 4.2. Secondary Outcomes

**Figure 5:**
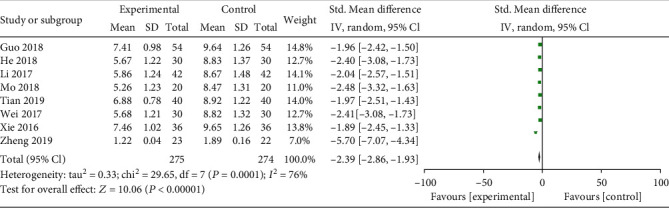
Forest plot for TCM syndromes and symptoms between combined and western alone medicine group.

**Figure 6:**
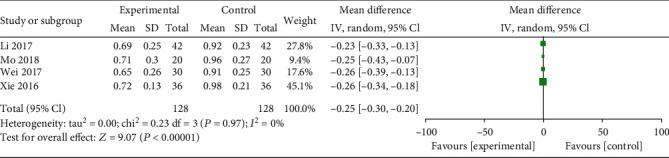
Forest plot for serum D-dimer level between combined and western alone medicine group.

**Figure 7:**
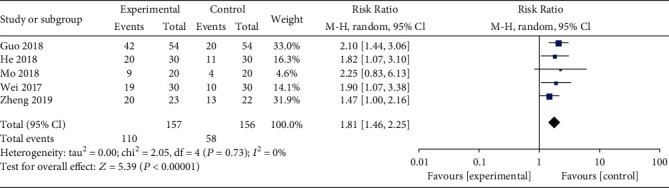
Forest plot for live birth rate between combined and western alone medicine group.

Eight studies assessed TCM syndromes and symptoms, five trials reported the incidence of live birth, and four trials evaluated serum D-dimer level. Compared with the western medicine alone group, patients treated with STP and western medicine had a lower TCM syndromes and symptoms (SMD: -2.39; 95% CI: -2.86, -1.93; *P* < 0.01, *I*^2^ = 76%, Figure 5) and serum D-dimer level (SMD: -0.25; 95% CI: -0.30, -0.20; *P* < 0.01, *I*^2^ = 0%, Figure 6), while higher incidence of live birth (RR: 1.81; 95% CI: 1.46–2.25; *P* < 0.01, *I*^2^ = 0%, Figure 7).

In the 12 included studies, only three trials reported on adverse events. No adverse effects of STP combined with western medicine group were identified in these trials, so there was insufficient data pooled to assess the safety of this intervention.

### 4.3. Publication Bias and Sensitivity Analysis

**Figure 8:**
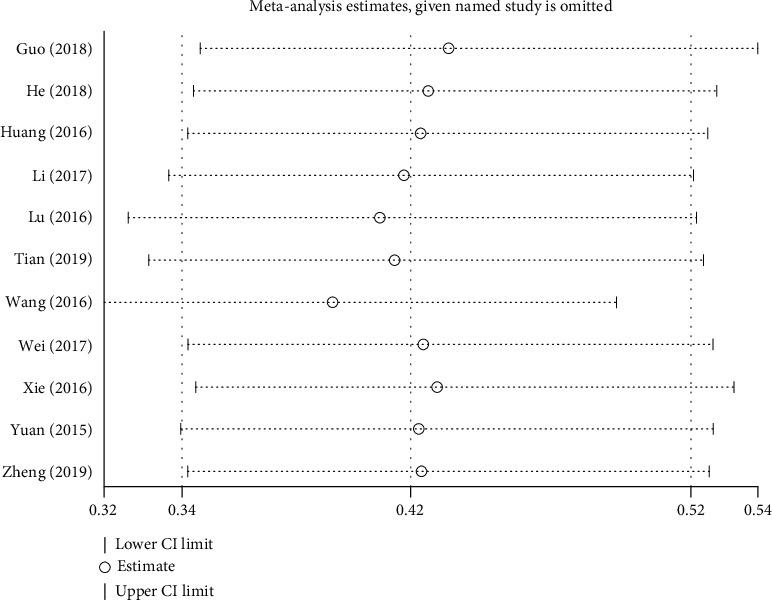
Sensitivity analysis for the early pregnancy loss rate.

Although the funnel plot for the early pregnancy loss rate was asymmetrically distributed, Egger's test showed no potential publish bias (*P* = 0.09). Sensitivity analysis was performed for the early pregnancy loss rate, and the effect estimate remained unchanged, which indicated the robustness of the pooled results (Figure 8).

### 4.4. GRADE Evaluation

We assessed the quality for the outcomes by GRADE evaluation. The quality of included studies was really low, and the “Risk of bias” was downgraded to “serious.” The *I*^2^ was high in the result of TCM syndromes and symptoms, and the “Inconsistency” was downgraded to “serious.” The result of GRADE evaluation for TCM syndromes and symptoms was low, while the quality for other outcomes was moderate. The overall results of GRADE evaluation are summarized in Table 3.

## 5. Discussion

### 5.1. Summary of Evidence

This meta-analysis demonstrated a quantitative estimates of the clinical efficacy and safety of cotreatment with STP and western medicine for the treatment of URSA by integrating outcomes from the 12 RCTs of 916 patients. The baseline of each study was consistent. Results from the meta-analysis showed that STP combined with western medicine can significantly reduce the early pregnancy loss incidence in the first trimester of pregnancy in women with URSA compared to the control group. Our meta-analysis also indicated that, compared with control group, STP combined with western medicine has the more significant effect on the live birth rate, TCM syndromes and symptoms, and serum D-dimer level. The safety of STP for the treatment of URSA remains unclear. However, due to poor methodological quality, the conclusion about beneficial effect and safety of STP combined with western medicine needed to be further verified in future studies.

### 5.2. Promising Complementary Therapy for URSA

In recent years, the incidence of URSA has increased causing a considerable negative life occurrence and may cause notable physical and psychological damage to couples who attempt to have a child due to the multifactorial, complex, and poorly understood pathogenesis of URSA [44]. There is currently no universally accepted standard treatment for URSA owing to it being identified by a diagnosis of exclusion and its complex molecular and cellular regulation [2].

Whether progesterone as the major method used in most included studies, or immunologic intervention and anticoagulant therapy administrated in a few included studies, there tends to have controversy and limitations such as adverse reactions like dizziness, nausea, and vomiting; potential risks include allergies, infectious diseases, and bleeding along with increasing financial burden [10]. New approaches for URSA will be needed and available in the future; Chinese herbal medicines, which regulate human body as a whole based on the four principal diagnostic methods of inspection, auscultation and olfaction, inquiry, and pulse-taking, are of great interest as complementary medicines [45]. Although it has the characteristics of complex composition and unclear mechanism, its comprehensive effect on disease, especially health care and prevention, is beyond the western medicine [46].

TCM has a unified medical theory for clinical diagnosis and treatment of URSA, which focuses on “Qi” and “blood” as the two basic elements of human physiology and believes that kidney-qi deficiency and blood stasis which can result in uterus pain and bleeding tendency are important in the pathology and mechanism of URSA [47]. Shoutai Pill, a well-known kidney-tonifying recipe in TCM for the prevention of miscarriage, is the basis of many other prescriptions [48]. In this meta-analysis, we found that STP combined with western medicine not only possess unique advantages in the prevention of miscarriage in patients with URSA but also has the potential to be highly effective at relieving symptoms and exhibiting good effects in activating blood flow and eliminating blood stasis by reducing the serum D-dimer level. It includes Chinese Dodder Seed as the main herb, supplemented by Chinese Taxillus Twig, Himalayan Teasel Root, and Donkey-hide Glue, playing the role of nourishing kidney and promoting generation of essence and blood to maintain a successful pregnancy preservation [28]. Among them, pharmacological studies have shown that the flavonoid components contained in Chinese Dodder Seed have estrogen-like function, which can improve reproductive endocrine function [49]; Alkaloids in Himalayan Teasel Root can inhibit uterine smooth muscle contraction and fight oxytocin [50]; flavonoid glycoside in Chinese Taxillus Twig has the action similar to progesterone which can supplement the insufficiency of endocrine function of patients [51]; and Donkey-hide Glue has a powerful function of enriching blood, exhibiting good effects in activating blood flow and eliminating blood stasis to significantly promote blood microcirculation [52], all of them affirm the important roles of Shoutai Pill in the treatment of URSA with no significant adverse effects found [53]. In summary, these herbal medicines can be used to improve the pregnancy outcome in the first trimester of pregnancy in women with URSA. Animal experiments reported that its treatment roles in the maintenance of normal pregnancy and in the mechanism of reducing adverse pregnancy outcomes may have multiple targets related to the regulation of immune conditions [54], the reduction of the apoptosis of trophoblast cells [55], and the improvement of hypothalamus-pituitary-gonadal axis [56]. However, valid evidence, including meta-analysis results, has yet to be needed for further recommendation.

## 6. Limitations

Although, we have comprehensively analyzed and evaluated all studies, it still has limitations that should be acknowledged. First, the included studies had low quality due to an unclear allocation concealment, selective bias, attrition bias, and blinding methods. Second, although we conducted an unbiased literature search without language restriction, all trials included in this review were conducted in China and were published in Chinese without relevant foreign experiments, which likely lead to a potential bias and therefore limit their representativeness. Third, few studies included have mentioned the adverse reactions during and after treatment. Meanwhile, no trial reported long-term follow-up, so the long-term safety of the intervention is still not well known. Fourth, the criteria for the outcome of each study were inconsistent. As a result, the evaluation had certain subjectivity and difference, which affected the accuracy and stability of the test.

## 7. Conclusion

In summary, our results showed that Shoutai Pill in combination with western medicine might increase the chances of a successful pregnancy in the first trimester of pregnancy for unexplained recurrent spontaneous abortion and may be beneficial for women with URSA as adjunct therapies without obvious adverse event in the experimental group. However, due to the relatively low quality of the included studies, we should be more cautious to promote this result. Future well-designed, multicenter, and large-sample clinical studies on evaluating the efficacy and safety of STP are needed to ensure the scientific, objective, and reliable conclusions of the research so as to make the results more convincing and provide more valuable information.

## Figures and Tables

**Table 1:** The basic characteristics of the included studies.

First author and year	Sample size (*n*)	Age (y)	Gestational age (d)	TCM syndrome differentiation	Times of abortions (*n*)	Definition of miscarriage	Definition of live birth	Intervention time	Intervention measures	Duration of intervention	Main outcomes
Guo 2018	T: 54 C: 54	T: 28.54 ± 3.03 C: 27.96 ± 2.85	T: NR C: NR	NR	T: 2.86 ± 0.71 C: 2.83 ± 0.75	Pregnancy loss before 16 weeks	Delivery of a live infant after 28 weeks	NR	T: modified STP (1/dose/day) + C C: dydrogesterone (10 mg, bid, po)	Until miscarriage or the 16th weeks	①②③
He 2018	T: 30 C: 30	T: 30.12 ± 2.22 C: 30.34 ± 2.07	T: 52.2 ± 3.7 C: 52.5 ± 3.3	Kidney deficiency and blood stasis	T: 2.6 ± 0.4 C: 2.7 ± 0.3	Pregnancy loss before 12 weeks	Delivery of a live infant after 37 weeks	Pregnancy confirmed by *β*-HCG	T: modified STP (1/dose/day) + C C: progesterone capsule (100 mg, bid, po)	3 months	①②③
Huang 2016	T: 31 C: 30	T: 27.77 ± 3.75 C: 26.47 ± 3.00	T: 45.68 ± 2.36 C: s	Spleen and kidney deficiency	T: 2.42 ± 0.62 C: s	Pregnancy loss before 12 weeks	NR	Pregnancy confirmed by ultrasound	T: modified STP (1/dose/day) + C C: progesterone injection (20 mg, qd, im)	14 days	①
Li 2017	T: 42 C: 42	T: 27.52 ± 3.69 C: 28.15 ± 3.62	T: 54.72 ± 4.96 C: 55.23 ± 4.65	NR	T: 2.32 ± 1.61 C: 2.52 ± 1.19	Pregnancy loss before 12 weeks	NR	Pregnancy confirmed by *β*-HCG	T: modified STP (1/dose/day) + C C: dydrogesterone (10 mg, bid, po)	NR	①③④
Lu 2016	T: 39 C: 37	T: 29.47 ± 2.05 C: 29.38 ± 2.16	T: 47.32 ± 3.17 C: 47.63 ± 3.51	NR	T: 2.83 ± 0.69 C: 2.94 ± 0.72	Pregnancy loss before 20 weeks	NR	Pregnancy confirmed by ultrasound	T: modified STP (1/dose/day) + C C: dydrogesterone (10 mg, bid, po)	Until miscarriage or the 16th weeks	①⑤
Mo 2018	T: 20 C: 20	T: 30.27 ± 3.41 C: 31.32 ± 3.26	T: NR C: NR	Kidney deficiency and blood stasis	T: 2.57 ± 1.33 C: 2.73 ± 1.12	NR	Delivery of a live infant after 37 weeks	Pregnancy confirmed by *β*-HCG	T: modified STP (1/dose/day) + C C: progesterone capsule (100 mg, bid, po) + dydrogesterone (10 mg, bid, po)	7 days for a course of treatment	②③④
Tian 2019	T: 40 C: 40	T: 27.88 ± 4.01 C: 28.11 ± 3.65	T: NR C: NR	NR	T: 3.01 ± 0.69 C: 2.88 ± 0.75	Pregnancy loss before 12 weeks	NR	Pregnancy confirmed by ultrasound	T: STP (1/dose/day) + C C: dydrogesterone (10 mg, q12h, po)	Until miscarriage or more than the 12th weeks	①③
Wang 2016	T: 75 C: 75	T: 31.7 ± 2.0 C: 31.4 ± 2.6	T: 47.5 ± 3.9 C: 47.2 ± 4.7	NR	T: NR C: NR	Pregnancy loss before 12 weeks	NR	Pregnancy confirmed by ultrasound	T: STP (1/dose/day) + C C: allogenic lymphocyte + dydrogesterone (10 mg, bid, po)	Until miscarriage or the 12th weeks	①
Wei 2017	T: 30 C: 30	T: 27.69 ± 3.52 C: 27.75 ± 3.43	T: 54.62 ± 4.95 C: 55.12 ± 4.76	Kidney deficiency and blood stasis	T: 2.67 ± 1.58 C: 2.59 ± 1.49	Pregnancy loss before 12 weeks	Delivery of a live infant after 37 weeks	Pregnancy confirmed by *β*-HCG	T: modified STP (1/dose/day) + C C: progesterone capsule (100 mg, bid, po)	NR	①②③④
Xie 2016	T: 36 C: 36	T: 30.5 ± 3.1 C: 30.7 ± 3.3	T: 52.1 ± 3.6 C: 52.4 ± 3.7	Kidney deficiency and blood stasis	T: 2.7 ± 0.3 C: 2.6 ± 0.2	Pregnancy loss before 12 weeks	NR	NR	T: modified STP (1/dose/day) + C C: dydrogesterone (10 mg, bid, po)	28 days	①③④⑤
Yuan 2015	T: 40 C: 40	T: 27.8 ± 5.5 C: 28.1 ± 5.7	T: NR C: NR	Kidney deficiency and blood stasis	T: 3.6 ± 1.2 C: 3.4 ± 1.1	Pregnancy loss before 12 weeks	NR	Pregnancy confirmed by ultrasound	T: modified STP (1/dose/day) + C C: dydrogesterone (10 mg, bid, po)	Until miscarriage or the 12th weeks	①⑤
Zheng 2019	T: 23 C: 22	T: 37.13 ± 4.45 C: 27.64 ± 2.88	T: NR C: NR	NR	T: 3.03 ± 1.58 C: 3.24 ± 1.24	Pregnancy loss before 28 weeks	Delivery of a live infant after 28 weeks	NR	T: STP (1/dose/day) + C C: progesterone injection (20 mg, qd, im) + dydrogesterone (10 mg, bid, po) + low-molecular weight heparin sodium (5000 iu, qd, h)	Until miscarriage or the 20th weeks	①②③

T: trial group; C: control group; NR: not reported; ①: the incidence of early pregnancy loss; ②: the incidence of live birth; ③: TCM syndromes and symptoms; ④: serum D-dimer level; ⑤: adverse events.

**Table 2:** Composition of prescription in the included studies.

References	Prescription	Composition of prescription
Guo et al. 2018, [32]	Modified STP	Chinese Dodder Seed 15 g, Chinese Taxillus Twig 15 g, Himalayan Teasel Root 10 g, *Eucommia ulmoides* 12 g, Donkey-hide Glue 12 g, *Cornus officinalis* 15 g, Chinese wolfberry 15 g, and Liquorice Root 6 g
He et al. 2018, [33]	Modified STP	Chinese Dodder Seed 30 g, Chinese Taxillus Twig 30 g, Himalayan Teasel Root 20 g, *Eucommia ulmoides* 20 g, Donkey-hide Glue 12 g, Chinese Angelica 12 g, *Salvia miltiorrhiza* 12g, White Paeony Root 10 g, Steamed Rehmannia Root 10 g, and Liquorice Root 6 g
Huang et al. 2016, [34]	Modified STP	Chinese Dodder Seed 20 g, Chinese Taxillus Twig 20 g, Himalayan Teasel Root 20 g, Donkey-hide Glue 10 g, *Radix codonopsis* 20 g, Largehead Atractylodes Rhizome 15 g, *Poria cocos* 10 g, and Liquorice root 5 g
Li et al. 2017, [35]	Modified STP	Chinese Dodder Seed 30 g, Chinese Taxillus Twig 30 g, Himalayan Teasel Root 20 g, *Eucommia ulmoides* 20 g, Donkey-hide Glue 12 g, Chinese Angelica 12 g, *Salvia miltiorrhiza* 12 g, White Paeony Root 10 g, Steamed Rehmannia Root 10 g, and Liquorice Root 6 g
Lu 2016, [36]	Modified STP	Chinese Dodder Seed 15 g, Chinese Taxillus Twig 15 g, Himalayan Teasel Root 9 g, Donkey-hide Glue 12 g, Chinese Wolfberry 15 g, *Cornus officinalis* 15 g, *Eucommia ulmoides* 12 g, *Salvia miltiorrhiza* 12 g, *Cinnamomi cortex* 9 g, and Liquorice Root 6
Mo et al. 2018, [37]	Modified STP	Chinese Dodder Seed 30 g, Chinese Taxillus Twig 30 g, Himalayan Teasel Root 20 g, Donkey-hide Glue 12 g, *Eucommia ulmoides* 20 g, Chinese Angelica 12 g, *Salvia miltiorrhiza* 12 g, White Paeony Root 10 g, Steamed Rehmannia Root 10 g, and Liquorice Root 6 g
Tian et al. 2019, [38]	STP	Chinese Dodder Seed 15 g, Chinese Taxillus Twig 15 g, Himalayan Teasel Root 10 g, and Donkey-hide Glue 12 g
Wang et al. 2016, [42]	STP	Chinese Dodder Seed 40 g, Chinese Taxillus Twig 20 g, Himalayan Teasel Root 20 g, and Donkey-hide Glue 20 g
Wei et al. 2017, [39]	Modified STP	Chinese Dodder Seed 30 g, Chinese Taxillus Twig 30 g, Himalayan Teasel Root 20 g, Donkey-hide Glue 12 g, *Eucommia ulmoides* 20 g, Chinese Angelica 12 g, *Salvia miltiorrhiza* 12 g, White Paeony Root 10 g, Steamed Rehmannia Root 10 g, and Liquorice Root 6 g
Xie et al. 2016, [40]	Modified STP	Chinese Dodder Seed 30 g, Chinese Taxillus Twig 30 g, Himalayan Teasel Root 20 g, Donkey-hide Glue 12 g, *Eucommia ulmoides* 20 g, Chinese Angelica 12 g, *Salvia miltiorrhiza* 12 g, White Paeony Root 10 g, Steamed Rehmannia Root 10 g, and Liquorice Root 6 g
Yuan et al. 2015, [41]	Modified STP	Chinese Dodder Seed 30 g, Chinese Taxillus Twig 30 g, Himalayan Teasel Root 20 g, Donkey-hide Glue 12 g, *Eucommia ulmoides* 20 g, Chinese Angelica 12 g, *Salvia miltiorrhiza* 12 g, White Paeony Root 10 g, Steamed Rehmannia Root 10 g, and Liquorice Root 6 g
Zheng 2019, [43]	STP	Chinese Dodder Seed 15 g, Chinese Taxillus Twig 15 g, Himalayan Teasel Root 15 g, and Root Donkey-hide Glue 15 g

STP: Shoutai Pill.

**Table 3:** The summary of GRADE evaluation.

Outcomes	Participants (studies)	RR/SMD (95% CI)	GRADE	Comments
The incidence of early pregnancy loss	876 (11 RCTs)	0.42 (0.34,0.52)	⨁⨁⨁◯ Moderate	“Risk of bias” was downgraded to “serious”
TCM syndromes and symptoms	549 (8 RCTs)	-2.39 (-2.86, -10.93)	⨁⨁◯◯ Low	“Risk of bias” was downgraded to “serious” “Inconsistency” was downgraded to “serious”
Serum D-dimer level	256 (4 RCTs)	-0.25 (-0.30, -0.20)	⨁⨁⨁◯ Moderate	“Risk of bias” was downgraded to “serious”
Live birth rate	313 (5 RCTs)	1.81 (1.46, 2.25)	⨁⨁⨁◯ Moderate	“Risk of bias” was downgraded to “serious”

RR: Risk Ratio; SMD: Standard Mean Difference; RCT: Random controlled trial.

## Data Availability

The data set supporting the results of this article is included within the article.
